# Supplementary material for: Down-Regulation of CYP3A4 by the KCa1.1 Inhibition Is Responsible for Overcoming Resistance to Doxorubicin in Cancer Spheroid Models
Source: Int J Mol Sci. 2023 Oct 27;24(21):15672. doi: 10.3390/ijms242115672 (PMC10648085; doi:10.3390/ijms242115672)
Supplement: Supplementary file 1 [file ijms-24-15672-s001.zip › ijms-2681915-supplementary.pdf]

Supplementary Materials: The following are available online at www.mdpi.com/xxx/s1

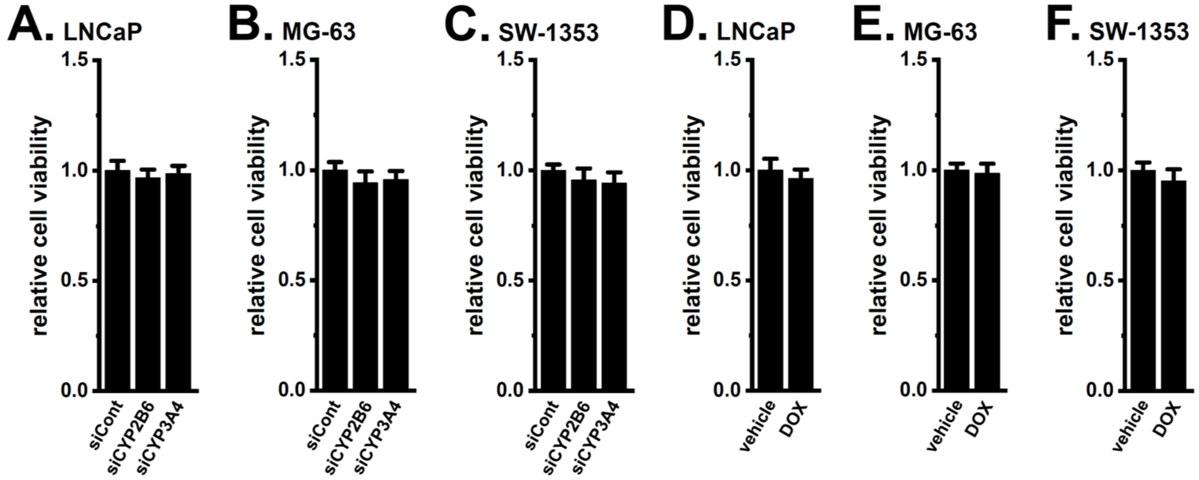

Figure S1. Effects of the siRNA-mediated inhibition of CYP2B6 and CYP3A4 without DOX treatment and DOX treatment without siRNA transfection on the viability of LNCaP, MG-63, and SW-1353 spheroid models using the WST-1 assay. A-C: Effects of transfection of negative control siRNA (siCont), human CYP2B6 siRNA (siCYP2B6), and human CYP3A4 siRNA (siCYP3A4) on the cell viability of LNCaP (A), MG-63 (B), and SW-1353 (C) spheroid models (at day 7 in LNCaP and at day 5 in MG-63 and SW-1353) (n = 5 for each). D-F: Effects of treatment with 1μM DOX for 48 hr on the cell viability of LNCaP (D), MG-63 (E), and SW-1353 (F) spheroid models (n = 5 for each). Cell viabilities in the siCont-transfected (A-C) and vehicle-treated (D-F) groups were expressed as 1.0.

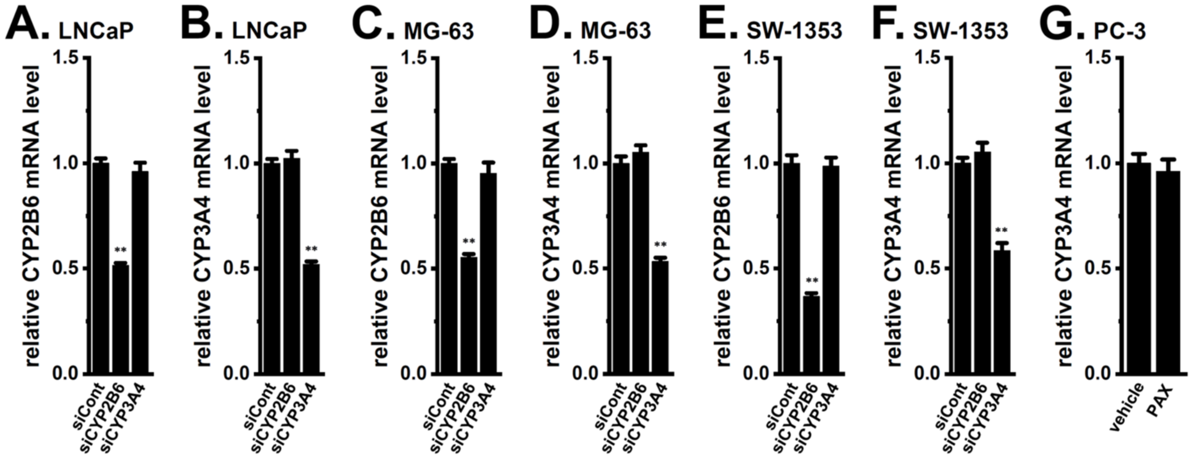

Figure S2. The transcriptional repression efficacy of siRNAs in cancer spheroid models and effects of a PAX treatment on CYP3A4 mRNA expression levels in the $K_{Ca}1.1$-negative PC-3 spheroid model. A-F: Real-time PCR examination of CYP2B6 (A,C,E) and CYP3A4 (B,D,F) transcripts in LNCaP (A,B), MG-63 (C,D), and SW-1353 (E,F) spheroid models transfected siCont, siCYP2B6, and siCYP3A4 for 72 hr (n = 4 for each). After normalization to ACTB mRNA expression levels, the mRNA expression levels of CYP2B6 and CYP3A4 in the siCont group were expressed as 1.0 (n = 4 for each). G: Real-time PCR examination of CYP3A4 transcripts in the vehicle- and PAX (10 μM)-treated PC-3 spheroid model for 12 hr. After normalization to ACTB mRNA expression levels, CYP3A4 mRNA expression levels in the vehicle control were expressed as 1.0 (n = 4 for each). **: $p < 0.01$ vs. siCont.

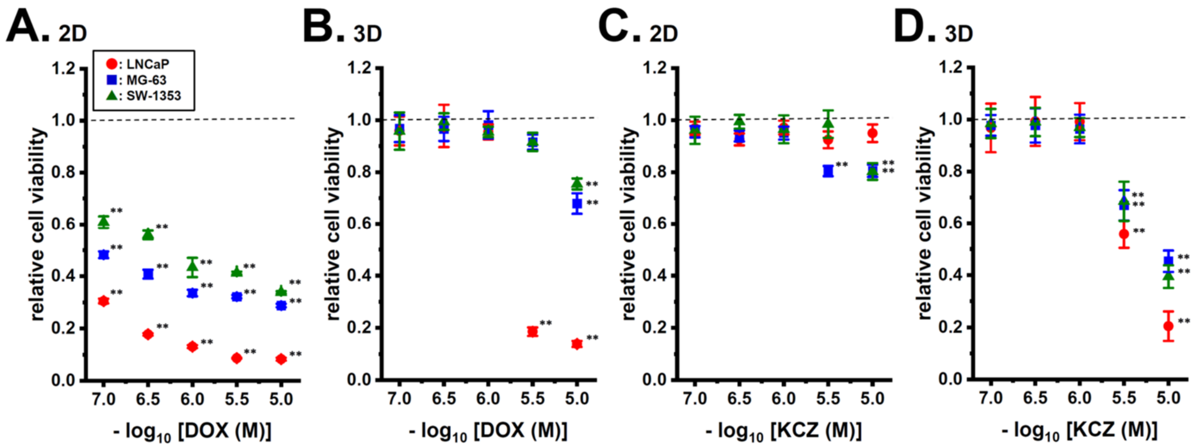

Figure S3. Concentration-response relationship of DOX and KCZ for the viability in 2D- and 3D-cultured LNCaP, MG-63, and SW-1353 cells. A,B: Effects of treatment with DOX (0.1, 0.3, 1.0, 3.0, and 10 μM) for 48 hr on the viability of 2D- (A) and 3D- (B) cultured LNCaP, MG-63, and SW-1353 cells. C,D: Effects of treatment with KCZ (0.1, 0.3, 1.0, 3.0, and 10 μM) for 48 hr on the viability of 2D- (C) and 3D- (D) cultured LNCaP, MG-63, and SW-1353 cells. The viability of the vehicle control-treated cells was expressed as 1.0. **: $p < 0.01$ vs. the vehicle control.

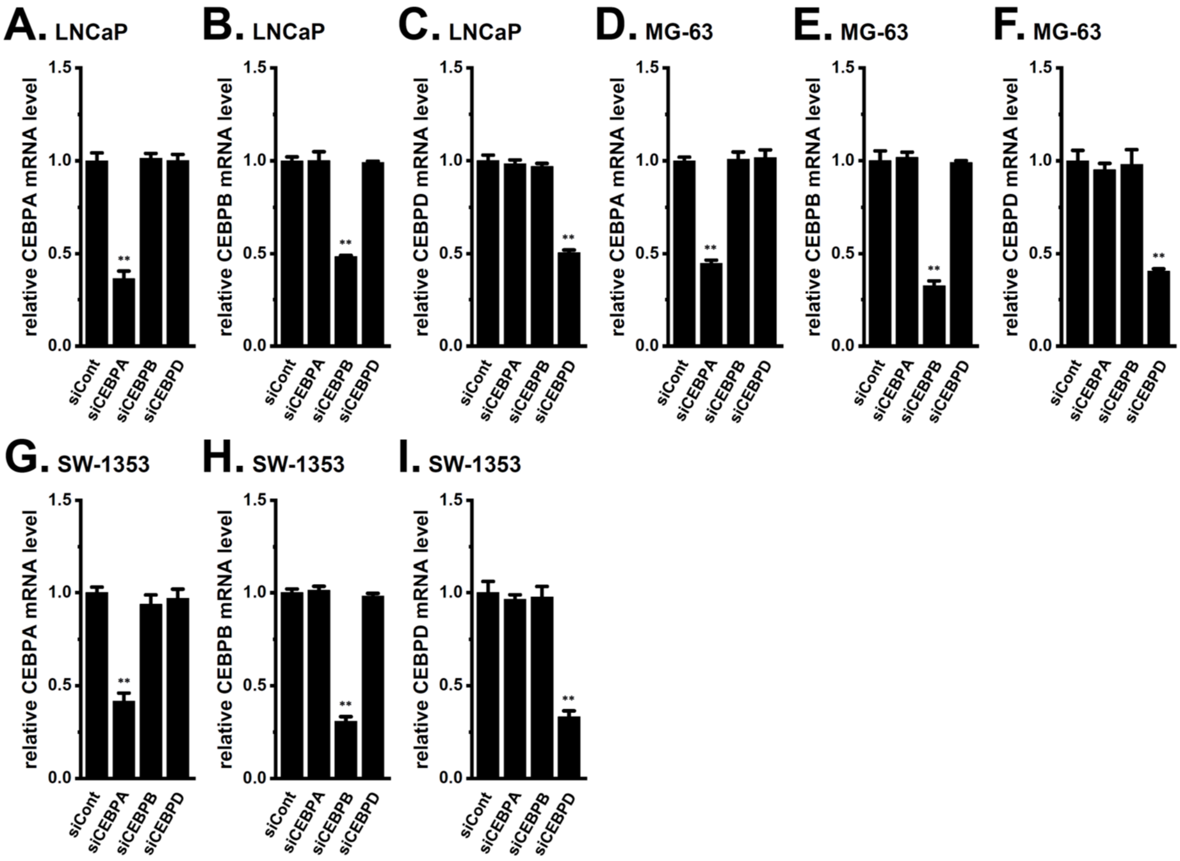

Figure S4. The transcriptional repression efficacy of siRNAs in cancer spheroid models. A-I: Real-time PCR examination of CEBPA (A,D,G), CEBPB (B,E,H), CEBPD (C,F,I) transcripts in LNCaP (A-C), MG-63 (D-F), and SW-1353 (G-I) spheroid models transfected siCont, siCEBPA, siCEBPB, and siCEBPD (n = 4 for each). After normalization to ACTB mRNA expression levels, the mRNA expression levels of CEBP isoforms in the siCont group were expressed as 1.0 (n = 4 for each). **: $p < 0.01$ vs. siCont.

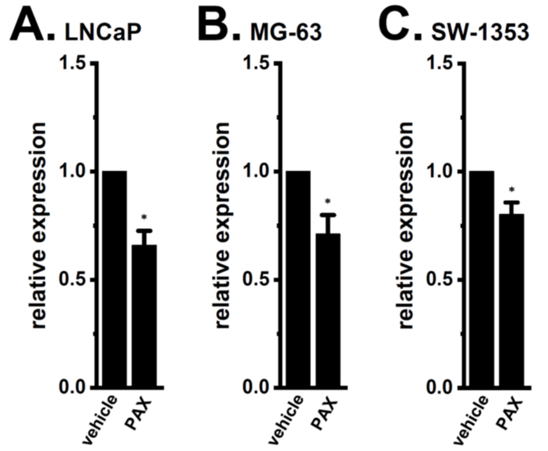

Figure S5. Effects of $K_{Ca}1.1$ inhibition on expression levels of total Akt (Akt) proteins in 3D spheroid models of LNCaP, MG-63, and SW-1353 cells. A-C: Protein lysates of LNCaP (A), MG-63 (B), and SW-1353 (C) spheroid models treated with the vehicle or 10 μM PAX for 2 hr were probed by immunoblotting with anti-Akt, and anti-ACTB antibodies (Fig. 7A,C,E). Akt signal in the vehicle-treated group (vehicle) was expressed as 1.0 (n = 4 for each). *: $p < 0.05$ vs. the vehicle control.

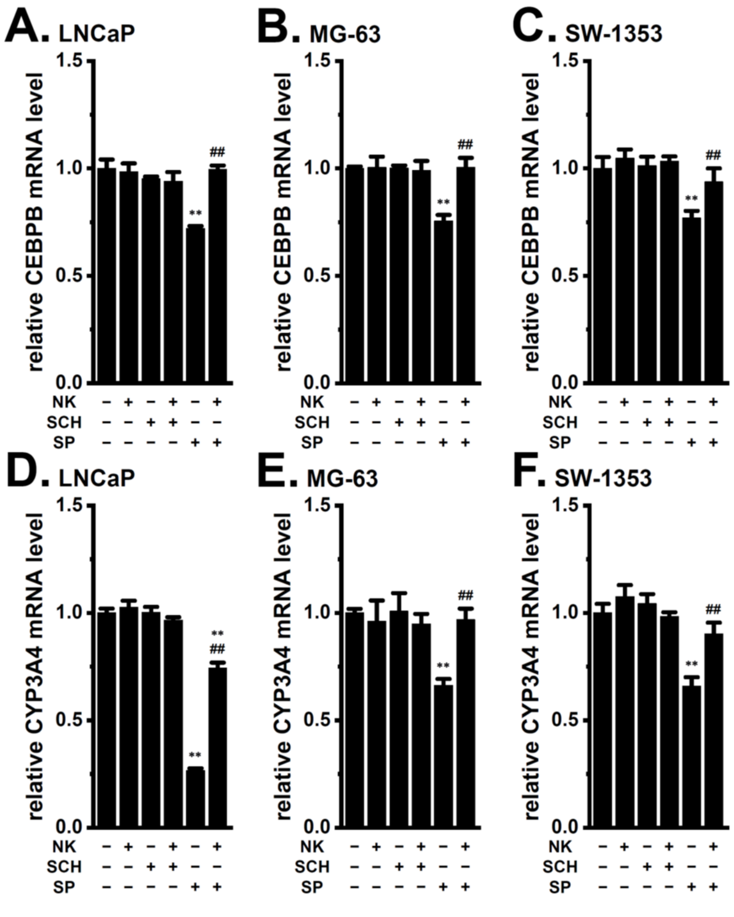

Figure S6. Effects of ERK and JNK inhibition on the expression levels of CEBPB and CYP3A4 transcripts in 3D spheroid models of LNCaP, MG-63, and SW-1353 cells. A-F: Real-time PCR examination of CEBPB (A-C) and CYP3A4 (D-F) in LNCaP (A,D), MG-63 (B,E), and SW-1353 (C,F) spheroid models treated (+) or untreated (−) with 100 μM NK252 (NK), 1 μM SCH772984 (SCH), and 1 μM SP600125 (SP) for 12 hr (n = 4 for each). After normalization to ACTB mRNA expression levels, CEBPB and CYP3A4 mRNA expression levels in the vehicle control (−/−/−) were expressed as 1.0. **: $P < 0.01$ vs. −/−/−; $^{\#\#}$: $P < 0.01$ vs. SP600125-treated group (−/−/+).

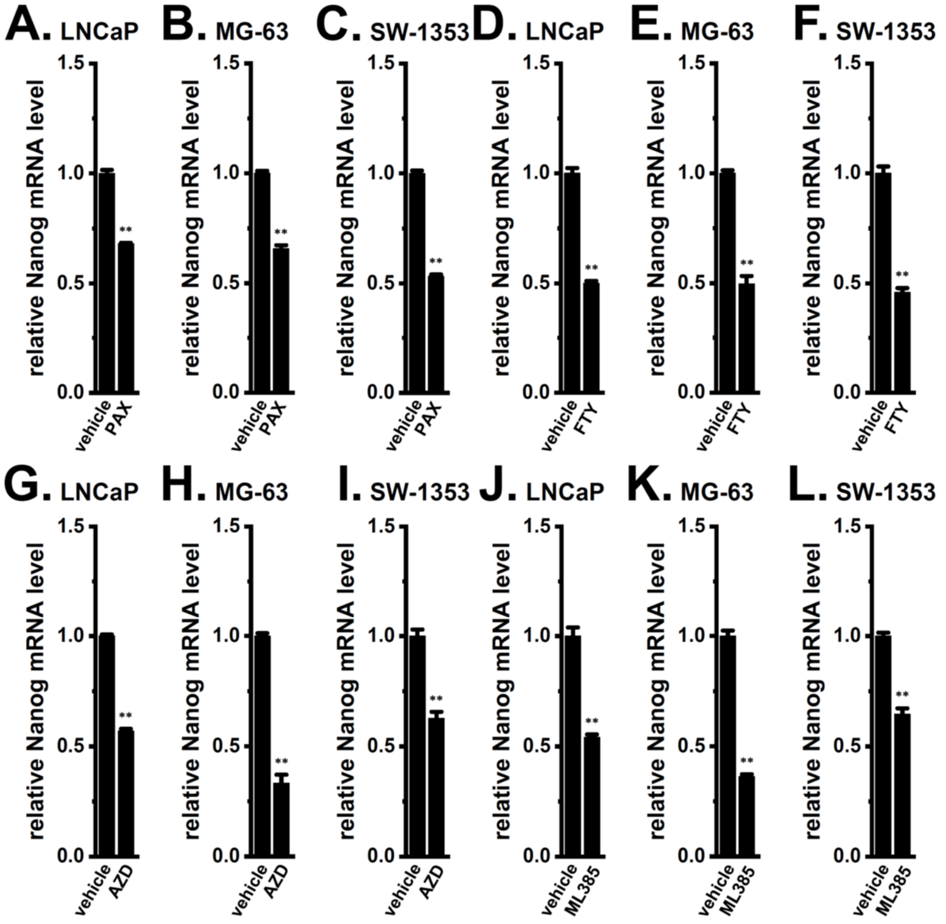

Figure S7. Effects of the inhibition of $K_{Ca}1.1$, Akt, and Nrf2 and the activation of PP2A on Nanog mRNA expression levels in LNCaP, MG-63, and SW-1353 spheroid models. A-L: Real-time PCR examination of Nanog in vehicle, 10 μM PAX (A-C), 5 μM FTY720-P (D-F), 2 μM AZD5363 (AZD) (G-I), 10 μM ML385 (J-L) -treated LNCaP (A, D, G, J), MG-63 (B, E, H, K), and SW-1353 (C, F, I, L) spheroid models for 12 hr (n = 4 for each). After normalization to ACTB mRNA expression levels, Nanog mRNA expression levels in the vehicle control were expressed as 1.0. **: $p < 0.01$ vs. the vehicle control.

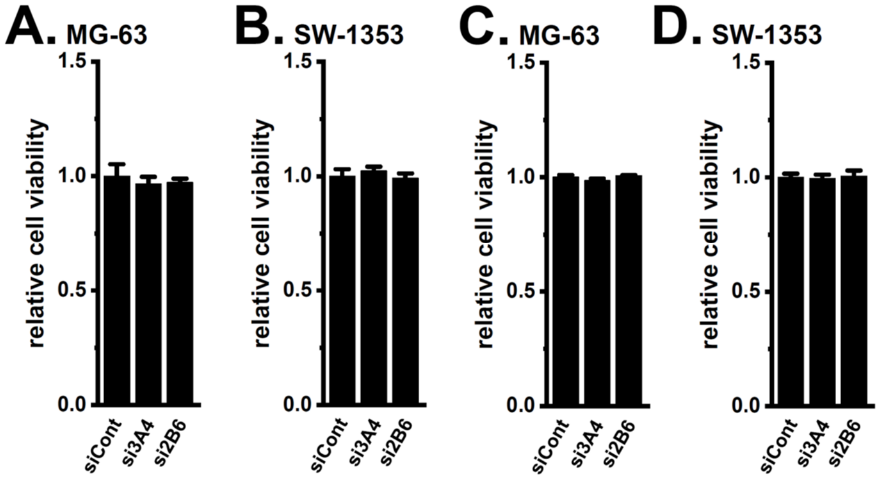

Figure S8. Effects of the siRNA-mediated inhibition of CYP3A4 and CYP2B6 on PTX and DTX resistance acquired by 3D spheroid models of MG-63 and SW-1353 cells. A,B: Effects of treatment with 100 nM PTX for 48 hr on the viability of MG-63 (A) and SW-1353 (B) spheroid models transfected with siCont, siCYP3A4 (si3A4), and siCYP2B6 (si2B6) using the WST-1 assay (n = 5 for each). C,D: Effects of treatment with 100 nM DTX for 48 hr on the viability of MG-63 (C) and SW-1353 (D) spheroid models transfected with siCont, siCYP3A4 (si3A4), and siCYP2B6 (si2B6) (n = 5 for each).

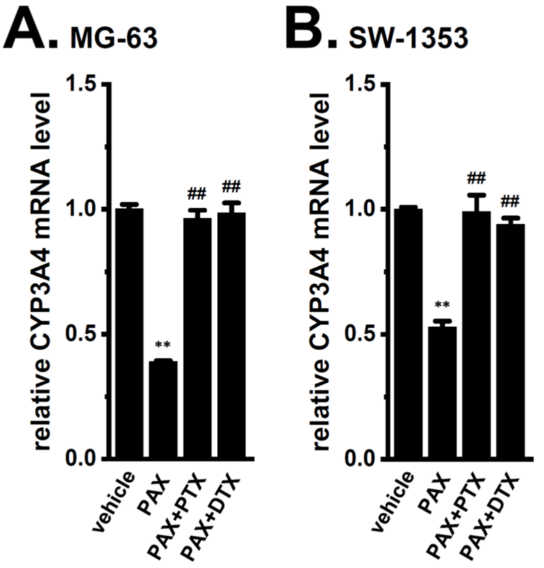

Figure S9. Effects of treatment with PTX and DTX for 12 hr on the $K_{Ca}1.1$ inhibition-induced down-regulation of CYP3A4 in MG-63 and SW-1353 spheroid models. A,B: Real-time PCR examination of CYP3A4 transcripts in PAX (10 μM), PAX plus PTX (100 nM), and PAX plus DTX (100 nM)-treated MG-63 (A) and SW-1353 (B) spheroid models for 12 hr. After normalization to ACTB mRNA expression levels, CYP3A4 mRNA expression levels in the vehicle control were expressed as 1.0 (n = 4 for each). **: $p < 0.01$ vs. the vehicle-treated group; $^{\#\#}$: $p < 0.01$ vs. the PAX-treated group.

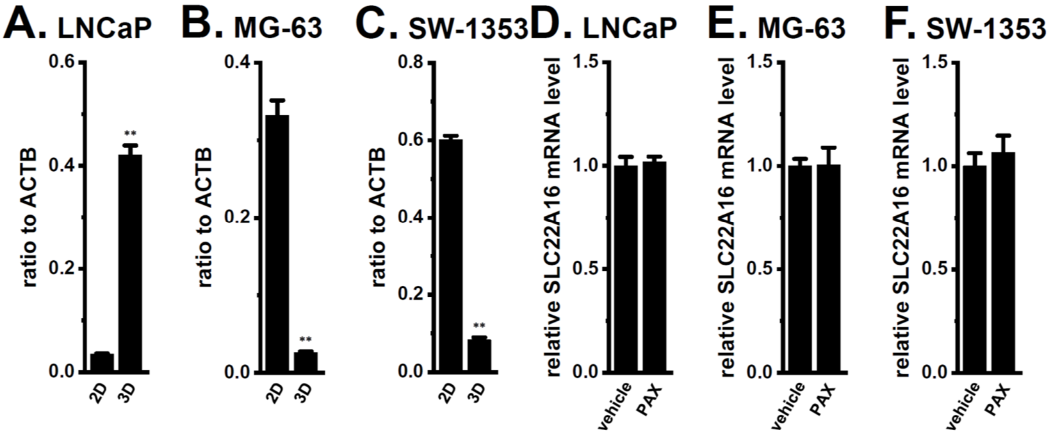

Figure S10. Comparison of SLC22A16 mRNA expression levels between 2D monolayers and 3D spheroid models of LNCaP, MG-63, and SW-1353 cells and effects of $K_{Ca}1.1$ inhibition on SLC22A16 mRNA expression in their spheroid models. A-C: Real-time PCR examination of SLC22A16 in 2D monolayers and 3D spheroid models of LNCaP (A), MG-63 (B), and SW-1353 (C) cells (n = 4 for each). D-F: Real-time PCR examination of SLC22A16 in LNCaP (D), MG-63 (E), and SW-1353 (F) spheroid models treated with vehicle and 10 μM PAX for 12 hr. After normalization to ACTB mRNA expression levels, the SLC22A16 mRNA expression levels in the vehicle control (vehicle) were expressed as 1.0. **: $p < 0.01$ vs. '2D'.
